# Integrative multi-omics analysis reveals cellular and molecular insights into gestational diabetes mellitus

**DOI:** 10.3389/fmolb.2026.1706588

**Published:** 2026-02-18

**Authors:** Niankun Chen, Shaole Shi, Huilin Xu, Shanshan Zhao, Letong Hong, Chumei Zeng, Yihong Huang, Lixia Shen, Dongyu Wang, Zilian Wang

**Affiliations:** Department of Obstetrics and Gynecology, The First Affiliated Hospital, Sun Yat-sen University, Guangzhou, China

**Keywords:** gestational diabetes mellitus, immune infiltration, key genes, Mendelian randomization, single-cell RNA sequencing

## Abstract

**Objective:**

Gestational diabetes mellitus (GDM) is a frequent pregnancy complication that increases short- and long-term risks for both mother and child. However, its underlying molecular mechanisms remain poorly understood. This study aims to unravel the molecular basis of GDM and explore potential therapeutic targets.

**Methods:**

We integrated genomic, transcriptomic, and single-cell RNA sequencing datasets to delineate cell-type-specific alterations in GDM. Candidate genes were prioritized using Mendelian randomization (MR), followed by quantitative PCR (qPCR) validation in placental samples. Pathway and immune-network analyses were performed to contextualize biological function.

**Results:**

Single-cell profiling showed marked remodeling of immune compartments in GDM, with prominent changes in monocytes and T-cell subsets. Two-sample MR prioritized 15 genes with putative causal links to GDM, including *BNIP3L*, *COMT*, *CTSB*, *LMNA*, and *SLC7A5*. qPCR further demonstrated significant differential expression of *CTSB*, *LMNA*, and *SLC7A5* between GDM and control placentas (human or mouse). Pathway enrichment implicated *CTSB* in immune regulation and metabolic processes, whereas *LMNA* and *SLC7A5* mapped to insulin resistance and glucose/amino-acid transport pathways. Immune-network analysis revealed significant correlations between these genes and immune mediators, supporting immune dysregulation as a contributor to GDM pathogenesis.

**Conclusion:**

This study provides a comprehensive analysis of the immune-metabolic landscape of GDM. Key genes identified in this study may serve as potential biomarkers and therapeutic targets for early diagnosis and personalized treatment of GDM. Further studies are warranted to elucidate the underlying mechanisms and develop targeted therapies for this disease.

## 1 Introduction

Gestational diabetes mellitus (GDM) is a state of hyperglycemia that is first diagnosed during pregnancy (Buchanan et al., 2012). Its global prevalence has risen over the past 4 decades (Jiang et al., 2022) and varies with diagnostic criteria, affecting an estimated 9%–25% of pregnancies (Alejandro et al., 2020). In a multi-center study, the prevalence ranged from 9% to 26% across 15 sites (Sacks et al., 2012). GDM confers substantial short-term risks, including preeclampsia and caesarean delivery for mothers, and macrosomia, neonatal hypoglycemia, and respiratory distress for infants (Plows et al., 2018; Simmons et al., 2024; Thornton et al., 2023). Long-term consequences include elevated risks of type 2 diabetes and cardiovascular disease in mothers and offspring, as well as obesity and metabolic syndrome in children (Hivert et al., 2024; Johns et al., 2018; Ye et al., 2022). Although genetic susceptibility, pregnancy-related insulin resistance, and β-cell dysfunction are implicated (Plows et al., 2018; Ray et al., 2024), the molecular determinants and cell-type-specific programs that drive GDM remain incompletely defined.

Single-cell RNA sequencing (scRNA-seq) enables the resolution of disease-relevant transcriptional programs at cellular granularity (Hong et al., 2020; Stuart and Satija, 2019; Tan et al., 2024), allowing dissection of immune and stromal compartments that may contribute to GDM pathophysiology. In parallel, Mendelian randomization (MR) provides a genetic framework for causal inference that is comparatively robust to confounding and reverse causation (Burgess et al., 2015; van de Vegte et al., 2020). Because non-coding variants often act through gene regulation, expression quantitative trait locus (eQTL) data, particularly from immune cell contexts, are widely used to connect variants to putative effector genes (Liu et al., 2025; McHenry et al., 2023; Wu et al., 2025). MR has yielded insights into the complications of diabetes, such as retinopathy and nephropathy (Tan et al., 2025; Wang et al., 2025), yet its application to GDM and integration with cell-type-aware transcriptomics remain limited.

Here, we integrate genomic, transcriptomic, and scRNA-seq datasets to interrogate the immune-metabolic architecture of GDM. We (i) map cell-type-specific alterations in peripheral and placental immune compartments using scRNA-seq, (ii) prioritize candidate effector genes for GDM via two-sample MR leveraging eQTL instruments, and (iii) validate key transcripts by quantitative PCR in placental tissue, complemented by pathway and immune-network analyses. This integrative strategy aims to nominate genetically informed biomarkers and therapeutic targets and to refine mechanistic hypotheses for early risk stratification and precision intervention in GDM.

## 2 Materials and methods

### 2.1 Data sources

Bulk RNA-seq. The series matrix for GSE263483 was downloaded from the NCBI Gene Expression Omnibus (GEO) and contains expression profiles from 16 placental samples (eight control and eight GDM samples).

Single-cell RNA-seq. Processed scRNA-seq data for GSE173193 were obtained from GEO, comprising four placental samples with complete single-cell expression profiles (two control and two GDM samples).

eQTL instruments (exposures). Whole-blood cis-eQTL summary statistics were retrieved from the eQTLGen consortium.

GDM outcome. Gestational diabetes genome-wide association study (GWAS) summary statistics were sourced from the FinnGen project (finn-b-GEST_DIABETES; 5,687 cases and 117,892 controls).

### 2.2 scRNA-seq preprocessing, QC, integration, and annotation

Raw/processed count matrices were analyzed in Seurat. Cells were filtered using multiple metrics: total unique molecular identifier (UMI) counts, number of detected genes (nFeature_RNA), and the proportions of mitochondrial and ribosomal transcripts. Outliers >3 median absolute deviations from the sample-wise median for any quality control (QC) metric were excluded. Putative doublets were identified and removed per sample using DoubletFinder (v2.0.4).

Data were normalized with LogNormalize (counts scaled to 10,000 per cell and log-transformed). Cell-cycle scores were computed (CellCycleScoring), highly variable genes were selected (FindVariableFeatures), and ScaleData was used to regress out mitochondrial/ribosomal percentages and cell-cycle effects. Linear dimensionality reduction used principal component analysis (PCA); principal components were chosen based on variance explained and elbow inspection. Batch effects were mitigated with Harmony on the selected PCs. Nonlinear embedding used UMAP.

Clusters were annotated by cross-referencing canonical markers (CellMarker and PanglaoDB) and literature, supplemented with automated labels from SingleR; manual curation resolved conflicts.

### 2.3 Cell–cell communication analysis

Intercellular ligand–receptor signaling was inferred with CellChat using normalized scRNA-seq data and the curated human ligand–receptor database. We quantified interaction strength (weight) and frequency (count) between annotated cell types/subtypes and summarized incoming/outgoing signaling to rank cell types by activity and influence.

### 2.4 Mendelian randomization (MR)

We implemented two-sample MR to estimate the causal effect of genetically proxied gene expression (exposure: eQTLGen whole blood) on GDM risk (outcome: FinnGen). For each gene, single-nucleotide polymorphisms (SNPs) associated with expression at genome-wide significance (P < 5e^−8^) were considered as candidate instruments and LD-clumped (R^2^ < 0.001; window 10,000 kb). Exposure and outcome alleles were harmonized; palindromic SNPs with ambiguous allele frequencies were excluded.

Primary causal estimates were obtained using inverse-variance weighted (IVW) meta-analysis, with MR-Egger, weighted median, and weighted mode complementary models to assess robustness. When only a single instrument was available, the Wald ratio was used.

### 2.5 MR sensitivity analyses

To evaluate robustness to influential variants, we performed leave-one-out analyses by iteratively excluding each SNP and recalculating the causal estimate, reporting point estimates and 95% CIs alongside the all-SNP estimate.

### 2.6 Immune cell deconvolution in bulk data

Immune infiltration was inferred from bulk expression (GSE263483) using CIBERSORT with the LM22 leukocyte signature (22 immune phenotypes). Only samples with deconvolution P < 0.05 were retained for interpretation. Pearson correlations were computed between candidate gene expression and inferred immune cell fractions.

### 2.7 Pathway enrichment analyses

Gene set enrichment analysis (GSEA). Samples were dichotomized by the median expression of the candidate gene(s). GSEA was performed against MSigDB v7.0 collections; significance was defined by FDR q < 0.05.

Gene set variation analysis (GSVA). GSVA scores were computed for Hallmark gene sets to profile pathway-level variation across samples.

### 2.8 Transcription factor (TF) regulon inference

TF motif enrichment was assessed using RcisTarget. For each gene set of interest (e.g., cluster markers or differentially expressed genes), area under the curve (AUC) scores were computed per motif-ranked gene list, and normalized enrichment scores (NES) were derived from the empirical AUC distribution to prioritize TFs.

### 2.9 Pseudotime analysis

Trajectory inference was performed with Monocle, ordering single cells along pseudotime to model state transitions within selected lineages. Differential gene dynamics along pseudotime were tested to identify state-dependent markers.

### 2.10 Sample collection and quantitative real-time PCR analysis

Human placenta: Placentas were collected at the First Affiliated Hospital of Sun Yat-sen University (Guangzhou, China): GDM (n = 8) and controls (n = 8). The study was approved by the institutional ethics committee ([2023]889); all participants provided written informed consent. GDM was diagnosed by a one-step 75-g, 2-h oral glucose tolerance test (OGTT) according to IADPSG criteria (Metzger et al., 2010). To minimize the impact of placental heterogeneity, all samples were consistently collected from the villous tissue in the central region of the placenta (midway between the maternal and fetal surfaces), avoiding the peripheral edges, fetal membranes, and maternal decidua. This sampling location is consistent with the tissue processing protocols used in the scRNA-seq and bulk RNA-seq datasets (GSE173193 and GSE263483) analyzed in this study.

Mouse GDM model: C57BL/6J mice (Sun Yat-sen University Animal Research Center) were housed under controlled conditions (22 °C ± 2 °C; 50%–60% humidity; 12-h light/dark; lights on 07:00) with *ad libitum* food/water. Four-week-old females were fed HFD (60% fat; D12492) or normal diet (10% fat; D12450B) for 6 weeks and then mated overnight (1:1). Pregnancy was confirmed by vaginal plug (E0.5). Mice were allocated to GDM (n = 8) or normal pregnancy (n = 8). A glucose tolerance test (GTT) (E9.5) and an insulin tolerance test (ITT) (E10.5) were performed as described (Sameshima et al., 2015; Yonezawa et al., 2012) to confirm the model. Placentas were harvested at E18.5 for RNA extraction. For the mouse model, whole placentas were harvested, and the central segments containing the labyrinth and junctional zones were prioritized for RNA extraction to ensure consistency with the functional analysis.

qRT-PCR: Total RNA was extracted with TRIzol (15596018; Invitrogen). cDNA was synthesized using PrimeScript RT Master Mix (RR036A; Takara), and quantitative PCR was performed on a CFX96 system using SYBR Premix Ex Taq (RR420A; Takara) following standard procedures (Wang et al., 2019). Primer sequences are listed in Supplementary Table S1.

### 2.11 Statistical analysis

Reliable Mendelian randomization (MR) analysis is based on three key assumptions: (1) correlation assumption (the instrumental variable is strongly associated with the exposure but not directly with the outcome), (2) independence assumption (the instrumental variable is not correlated with confounding factors), and (3) exclusivity assumption (the instrumental variable influences the outcome solely through the exposure; the presence of pleiotropy occurs when the IV affects the outcome through other pathways). The Wilcoxon test and unpaired t-test were performed for statistical comparisons, and the chi-squared test was used for categorical variables, as appropriate. All analyses in this study were performed using R software (version 4.3.0). Statistical tests were two-sided, with a p-value <0.05 considered statistically significant.

## 3 Results

### 3.1 Construction of the single-cell transcriptomic landscape

**FIGURE 1:**
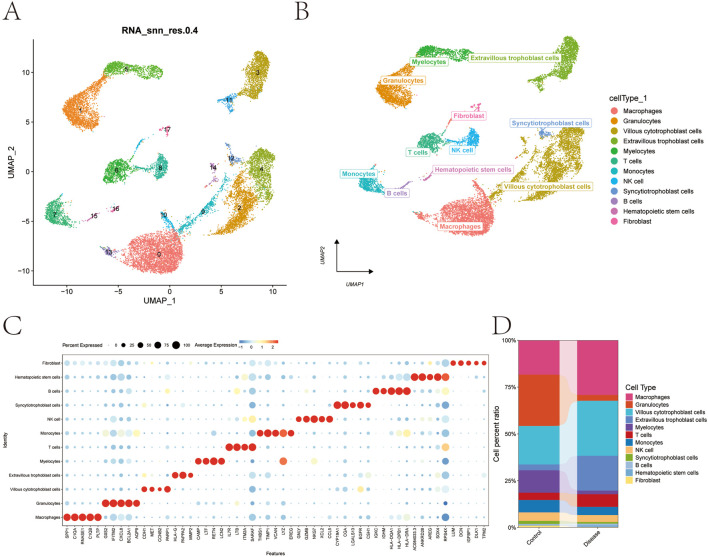
Cell subpopulation identification and annotation. **(A)** UMAP plot showing the identification of 18 distinct cell subpopulations after dimensionality reduction. **(B)** Cell annotation of the 18 subpopulations using known cell markers, classified into 12 different cell types: macrophages, granulocytes, villous cytotrophoblast cells, extravillous trophoblast cells, myelocytes, T cells, monocytes, NK cells, syncytiotrophoblast cells, B cells, hematopoietic stem cells, and fibroblasts. **(C)** Bubble plot of classical markers for the 12 cell types. **(D)** Bar plot illustrating the cell-type proportions across different subgroups.

To characterize the cellular heterogeneity of the placental microenvironment in GDM, we performed scRNA-seq and implemented a rigorous quality control pipeline. After filtering cells with low feature counts and applying MAD-based outlier removal, doublets were excluded via DoubletFinder, yielding 17,316 high-quality cells (Supplementary Figure S1A,B). We identified 2,000 highly variable genes (Supplementary Figure S1C) and, following Harmony batch correction, UMAP visualization revealed 18 transcriptionally distinct clusters (Supplementary Figure S1D–F; Figures 1A). Using canonical markers and SingleR assistance, these were consolidated into 12 major cell types, including trophoblasts, T cells, monocytes, and macrophages (Figures 1B,C). The group-wise composition analysis provided an initial overview of the cellular shifts in GDM placentas (Figure 1D).

### 3.2 Cell-type-specific functional activity and intercellular communication

**FIGURE 2:**
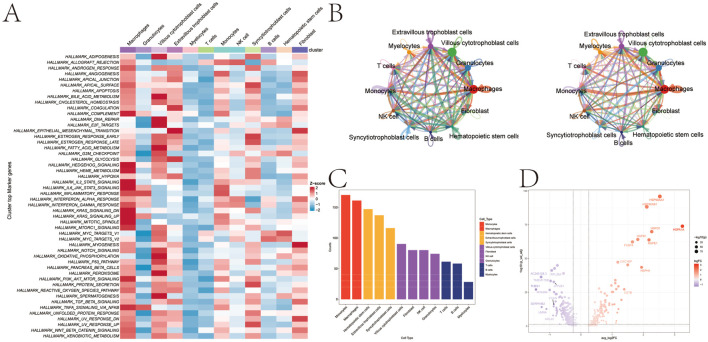
Pathway activity and cell communication analysis. **(A)** Heatmap showing the pathway activity across different cell subtypes, based on AUCell analysis. **(B)** Ligand–receptor interaction network between cell subtypes (the figure on the left shows the presence or absence of interactions, while the figure on the right shows the strength or significance of these interactions). **(C)** Interaction strength of monocytes with other cell types. **(D)** Volcano plot displaying differentially expressed genes in monocytes.

To further explore the functional states of these cells, we quantified single-cell pathway activity using AUCell. The results indicated relatively low activity for most metabolism-related pathways in myelocytes and T cells (Figure 2A). We then employed CellChat to infer the intercellular communication network, which revealed a dense signaling web among placental cells (Figure 2B). Notably, monocytes emerged as the central hub, exhibiting the highest number and strength of both incoming and outgoing interactions (Figure 2C). Differential expression analysis within these high-activity monocytes identified 422 genes that were significantly dysregulated (|avg_log_2_FC| > 0.25; P < 0.05; Figure 2D), suggesting their critical role in GDM-associated inflammation.

### 3.3 Causal prioritization of GDM effector genes via Mendelian randomization

**FIGURE 3:**
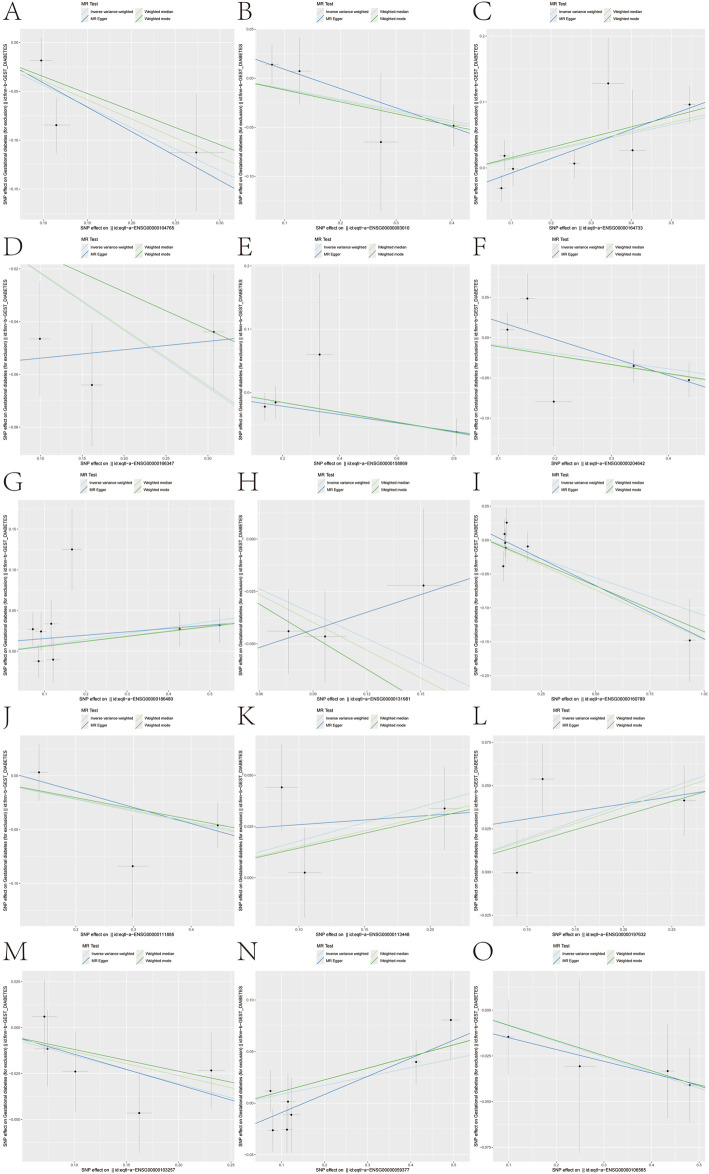
Mendelian randomization analysis for GDM-associated genes. **(A–O)** Forest plots showing Mendelian randomization results for 15 gene–eQTL relationships significantly associated with GDM outcomes (IVW p-value <0.05). Genes include *BNIP3L*, *COMT*, *CTSB*, *CYB5A*, *FCER1G*, *HLA-F*, *INSIG1*, *LGALS3*, *LMNA*, *MAN1A1*, *PDE4D*, *SERPINB2*, *SLC7A5*, *TBXAS1*, and *TMEM176B*, with specific odds ratios and confidence intervals for each gene.

**FIGURE 4:**
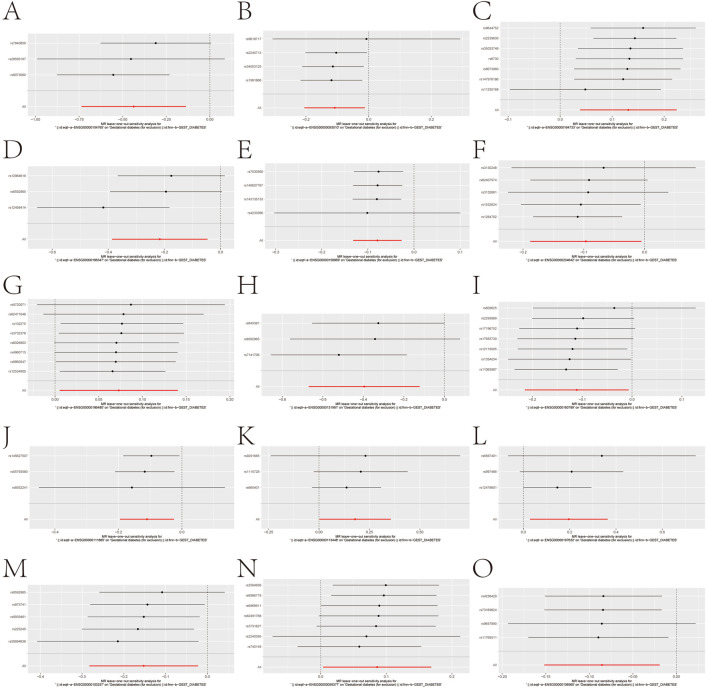
Sensitivity analysis of causal relationships. **(A–O)** Sensitivity plots showing the robustness of the 15 gene causal relationships by testing the removal of individual SNPs.

To circumvent observational confounding, we applied two-sample MR using eQTLGen whole-blood instruments and FinnGen GDM outcomes (5,687 cases; 117,892 controls). Evaluating 361 gene–outcome pairs, we identified 15 genes with nominal causal associations (P < 0.05) to GDM (Figures 3A–O). Specifically, genetically proxied higher expression of *BNIP3L* (OR 0.646), *COMT* (0.897), *CYB5A* (0.805), *FCER1G* (0.924), *HLA-F* (0.908), *LGALS3* (0.673), *LMNA* (0.895), *MAN1A1* (0.896), *SLC7A5* (0.859), and *TMEM176B* (0.918) was associated with reduced GDM risk. Conversely, *CTSB* (OR 1.140), *INSIG1* (1.076), *PDE4D* (1.196), SERPINB2 (1.217), and TBXAS1 (1.090) were identified as risk factors. Leave-one-out analyses confirmed the stability of these associations (Figures 4A–O).

### 3.4 Validation of key genes and immune infiltration remodeling

**FIGURE 5:**
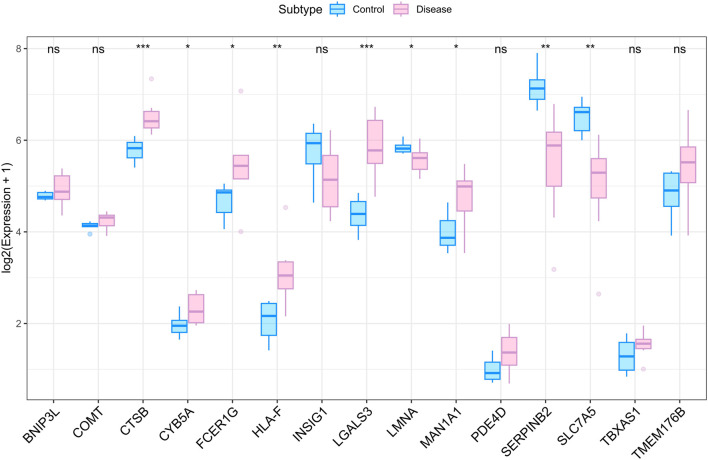
Gene trend validation and immune infiltration analysis. (A) Differential expression analysis of 15 genes identified in the transcriptome.

**FIGURE 6:**
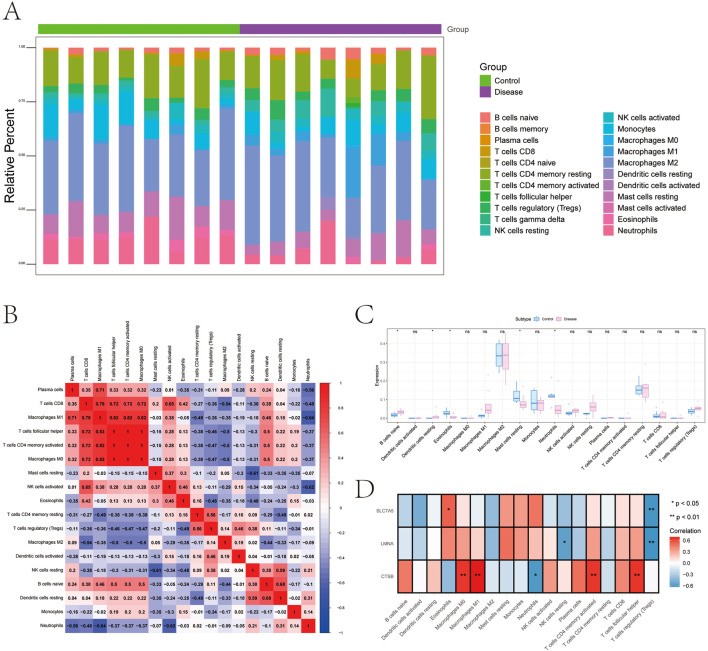
Immune infiltration and gene correlations with immune cells. **(A)** Distribution of immune infiltration levels in the disease and control groups. **(B)** Immune cell correlations across different cell types in the disease and control groups. **(C)** Comparison of immune cell infiltration levels between the disease and control groups. **(D)** Correlation analysis between key genes (*CTSB*, *LMNA*, and *SLC7A5*) and immune cell types.

The expression directions of our top candidates, *CTSB*, *LMNA*, and *SLC7A5*, were supported by external validation in the GSE263483 dataset (Figure 5A). Given the role of the microenvironment, CIBERSORT deconvolution revealed that GDM placentas undergo significant immune remodeling, characterized by altered proportions of naïve B cells, eosinophils, and neutrophils (Figures 6A–C). Correlation analyses linked our key genes to these immune shifts: *CTSB* correlated positively with CD4^+^ memory T cells and M1 macrophages, while *LMNA* and *SLC7A5* negatively associated with Tregs and natural killer (NK) cells (Figure 6D). Furthermore, these genes exhibited strong correlations with various immune modulators, including chemokines (e.g., *CTSB* with CXCL9), receptors (CCR5), and immunoinhibitors (Supplementary Figure S2A-E), reinforcing their role in immune-metabolic regulation.

### 3.5 Pathway enrichment and mechanistic insights (GSEA/GSVA)

**FIGURE 7:**
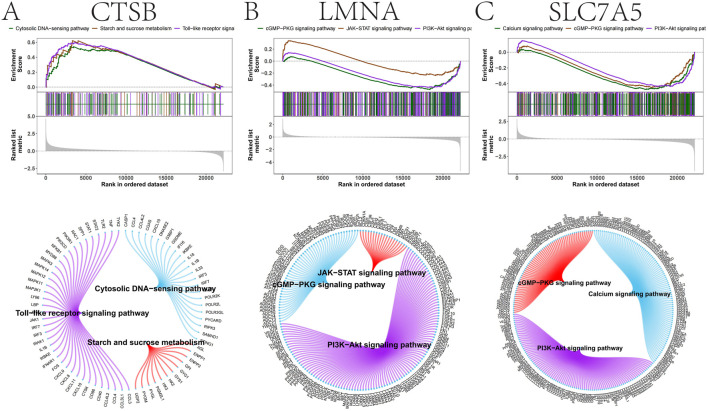
GSEA of key gene enrichment in signaling pathways. **(A)** Pathway enrichment analysis for *CTSB*. **(B)** Pathway enrichment analysis for *LMNA*. **(C)** Pathway enrichment analysis for *SLC7A5*.

To probe the mechanisms linking these genes to GDM, we stratified samples by key gene expression. GSEA indicated that *CTSB* aligns with innate immune programs (Toll-like receptor and cytosolic DNA-sensing) and carbohydrate metabolism (Figure 7A). In contrast, *LMNA* and *SLC7A5* mapped to core metabolic signaling nodes, including the PI3K–AKT and JAK–STAT pathways (Figures 7B,C). GSVA corroborated these findings, linking *CTSB* to epithelial-mesenchymal transition and *LMNA*/*SLC7A5* to adipogenesis and heme metabolism (Supplementary Figure S3A-C). Collectively, these results suggest that *CTSB* primarily drives inflammatory activation, while *LMNA* and *SLC7A5* modulate nutrient-sensing and insulin signaling axes.

### 3.6 Regulatory network and disease–gene integration

**FIGURE 8:**
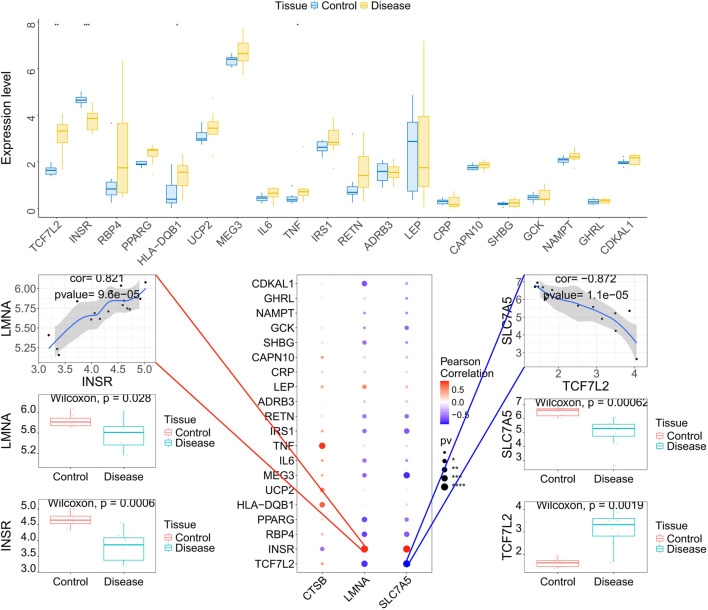
Transcription factor regulatory network and relationship between key genes and disease-associated genes. **(A)** Enrichment analysis of transcription factor motifs for the key genes. **(B)** Corresponding transcription factors associated with the enriched motifs for the key genes. **(C)** Correlation between key genes and disease-related regulatory genes from the GeneCards database. Expression differences of the top 20 disease-related genes with the highest Relevance* scores were analyzed, and their correlation with the key genes was assessed.

Construction of a transcription factor (TF) regulatory network identified multiple upstream drivers, with the motif cisbp_M6146 exhibiting the highest enrichment (NES = 9.47; Figure 8). We also integrated these candidates with high-relevance GDM genes from GeneCards. The key genes not only showed cell-type-specific expression patterns (Supplementary Figure S4A,B) but also formed dense co-expression networks with established disease genes (Supplementary Figures S5–S7). Notably, *LMNA* was strongly positively correlated with *INSR* (r = 0.821), while *SLC7A5* correlated negatively with *TCF7L2* (r = −0.872) (Figure 8), placing our candidates within the known pathogenic framework of GDM.

### 3.7 Spatiotemporal dynamics and immune-metabolic activity

**FIGURE 9:**
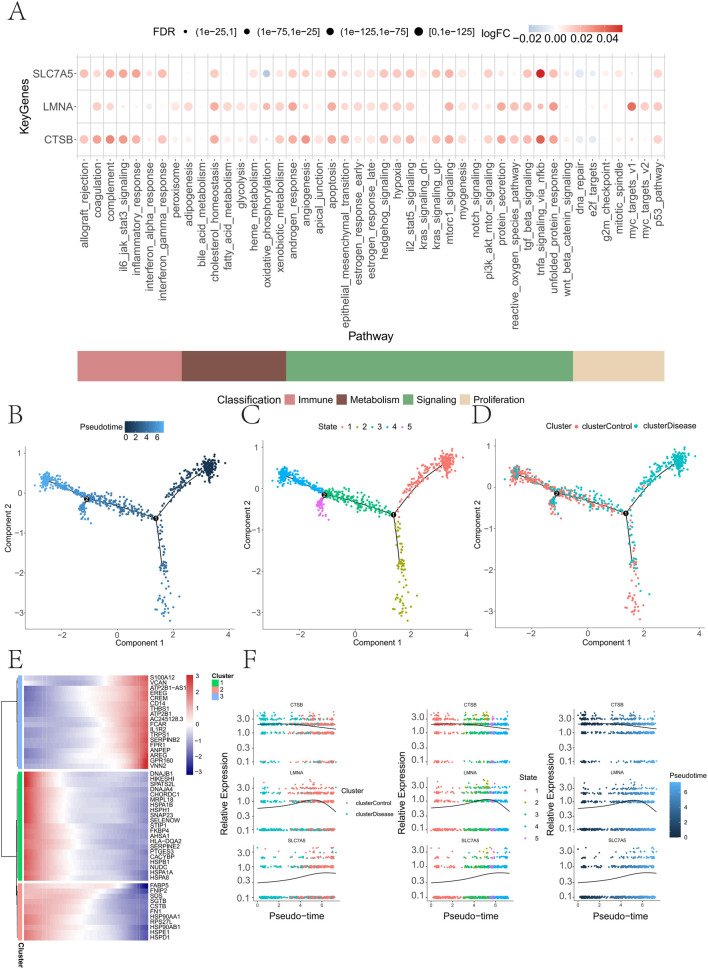
Immune metabolism-related pathway activity in single cells, pseudotime analysis of cell differentiation trajectory, and key gene expression. **(A)** Bubble plot showing the expression differences of key genes (*LMNA*, *CTSB*, and *SLC7A5*) in immune metabolism-related pathways. **(B–D)** Pseudotime-based visualization of cell differentiation. **(E)** Differential gene expression patterns along pseudotime, grouped by the largest expression changes. **(F)** Expression of key genes (*CTSB*, *LMNA*, and *SLC7A5*) along the pseudotime trajectory.

AUCell scoring further demonstrated that cells with elevated expression of key genes exhibited higher activity in androgen response and TNF-α signaling via NF-κB (Figure 9A). Monocle-based pseudotime analysis revealed a distinct trajectory in which GDM cells predominated at earlier states, whereas control cells progressed toward later states (Figures 9B–D). Dynamic gene clustering identified early (e.g., DNAJB1) and late (e.g., S100A12) programs (Figure 9E). Along this pseudotime, *CTSB* expression decreased, *LMNA* showed a transient increase, and *SLC7A5* increased monotonically (Figure 9F), highlighting their roles as dynamic markers of cellular transition in the GDM placenta.

### 3.8 Experimental validation in human and murine models

**FIGURE 10:**
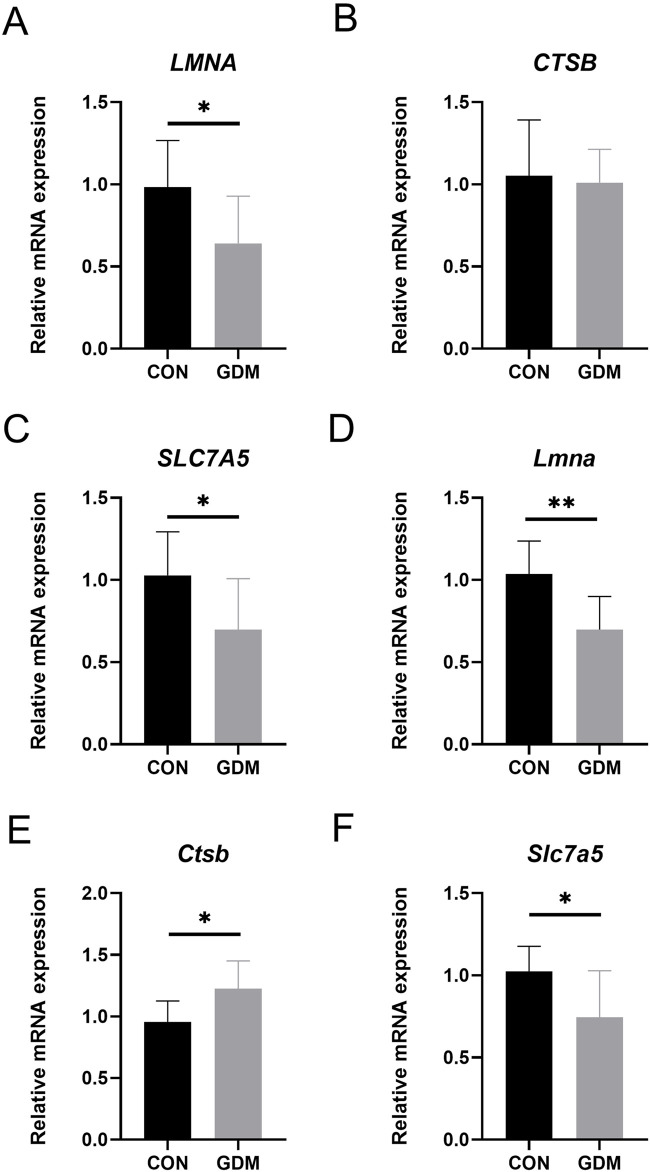
mRNA expression levels of key genes in human and mouse placentas. **(A–C)** represent the mRNA expression levels of *LMNA*, *CTSB*, and *SLC7A5*, respectively, in human placentas from the GDM and normal control groups. **(D–F)** represent the mRNA expression levels of *LMNA*, *CTSB*, and *SLC7A5*, respectively, in mouse placentas from the GDM and normal control groups. * indicates P < 0.05; ** indicates P < 0.01.

To confirm our findings, we quantified mRNA levels in placental tissues. The GDM mouse model was successfully established, as evidenced by GTT and ITT results (Supplementary Figure S8). In human placentas, *LMNA* and *SLC7A5* were significantly downregulated in GDM vs. controls (P < 0.05). While *CTSB* showed no significant difference in human samples, its expression was significantly elevated in GDM mouse placentas (P < 0.05; Figure 10). These results provide *in vivo* evidence supporting the involvement of these prioritized genes in GDM pathology.

## 4 Discussion

GDM is a significant health concern during pregnancy, yet its molecular mechanisms remain incompletely understood. In this study, we combined single-cell transcriptomics with Mendelian randomization (MR) analysis to investigate the potential molecular mechanisms underlying GDM, identify key genes, and explore the immune-metabolic pathways they regulate. Our findings provide new insights into single-cell immune cell infiltration characteristics, differential gene expression, and causal relationships between gene expression and disease outcomes, contributing to a deeper understanding of GDM pathophysiology. These findings may imply future precision medicine strategies for diagnosis and intervention.

First, single-cell transcriptomic analysis revealed cellular heterogeneity in the blood and placenta of GDM patients. By annotating 18 distinct cell subpopulations, we identified altered immune cell subpopulation activities (e.g., monocytes and T cells) in GDM patients, with particularly noticeable changes in the signaling interactions between monocytes and other immune cells. This provides direct evidence for the altered immune microenvironment in pregnancy and suggests a potential role for immune cells in GDM pathogenesis. In particular, immune cell infiltration analyses highlighted significant changes in the activation and function of monocytes, T cells, and macrophages in GDM, suggesting their involvement in the immune-mediated progression of the disease. Cell–cell communication network analysis further highlighted the prominent role of monocytes in the GDM group, with increased interaction with other cell types, suggesting that monocytes may be crucial in the initiation and progression of GDM. Immune cell infiltration analysis confirmed the significant involvement of T cells and macrophages in the disease, aligning with previous reports that emphasize the role of immune cell dysfunction and placental disturbances in GDM development (Chen et al., 2023; Chen J et al., 2024; Du R et al., 2022; McElwain et al., 2021).

Several studies have employed genetic association studies and transcriptomic analyses to explore the molecular mechanisms of GDM (Huang et al., 2024; Pervjakova et al., 2022; Powe and Kwak, 2020; Yang et al., 2021). Our analysis confirmed changes in gene expression in macrophages, monocytes, and trophoblast cells in GDM, consistent with prior research emphasizing the involvement of immune cells and placental dysfunction in GDM (Desoye and Hauguel-de, 2007; Fagninou et al., 2022; Ji et al., 2024). In line with recent studies, distinct placental gene expression signatures have been identified between GDM subtypes (GDMA1 and GDMA2), providing further insights into the differential molecular mechanisms associated with each GDM subtype (Barrozo et al., 2025). Moreover, a comprehensive pan-organ transcriptomic atlas has elucidated the maternal and fetal metabolic and immune landscapes in GDM, highlighting the complex interactions between maternal metabolism and immune responses in both the placenta and fetal tissues (Ni and Nanan, 2026). However, our study further reveals a complex cell–cell communication network and immune cell infiltration pattern, offering new perspectives on the immune-mediated mechanisms of GDM.

We also applied Mendelian randomization to systematically assess the causal relationship between blood gene expression and GDM risk using large-scale genotype-disease outcome data. We identified 15 key genes (including *BNIP3L*, *COMT*, and *CYB5A*) and confirmed their causal associations with GDM. Specifically, genes such as *BNIP3L*, *COMT*, and *CYB5A* were associated with lower GDM risk, whereas *CTSB*, *INSIG1*, and *SERPINB2* were associated with higher GDM risk. These genes are involved in metabolic regulation and may contribute to GDM pathogenesis by modulating immune responses or cellular signaling pathways. Notably, the consistent differential expression of *CTSB*, *LMNA*, and *SLC7A5* across transcriptomic data and MR analysis provides critical insights into the molecular mechanisms of GDM. We further analyzed the human and mouse placentas from the GDM group using qPCR, confirming significant differences in *CTSB*, *LMNA*, and *SLC7A5* expression in the GDM group.

Functional pathway analyses revealed that *CTSB*, *LMNA*, and *SLC7A5* may influence GDM progression via multiple signaling pathways. *CTSB* was enriched in immune response and metabolic regulation pathways, including the Toll-like receptor signaling pathway and DNA-sensing pathways, highlighting its pivotal role in immune modulation and metabolism (Chen Y et al., 2024; Kawasaki and Kawai, 2014). *LMNA*, which is associated with the PI3K–AKT signaling pathway, is implicated in the regulation of cell growth and metabolism, potentially impacting GDM pathophysiology (Miao et al., 2022; Torres-Torres et al., 2024; Yu and Cui, 2016). One study showed that LMNA is associated with trophoblast senescence in preeclampsia (Xiao et al., 2024), suggesting that the pathological mechanism linking GDM to a predisposition to preeclampsia may involve *LMNA*. *SLC7A5* plays a role in calcium and PI3K–AKT signaling pathways, which are tightly linked to insulin resistance and glucose metabolism (Gao et al., 2015; Miao et al., 2022; Ramasubbu and Devi, 2023). Immune modulator analysis indicated that CTSB significantly correlates with several immune factors (e.g., CXCL9 and CCR5). In contrast, *LMNA* and *SLC7A5* are negatively correlated with immune suppressors such as PDCD1LG2, offering new insights into the role of the immune system in GDM.

Trajectory analysis of cell differentiation further revealed that cells in the GDM group predominantly localized to the early stages of differentiation, whereas the cells in the control group tended to cluster in the later stages. This could reflect a delay in the differentiation process in GDM patients, suggesting that cellular fate regulation may be disrupted during GDM progression. Gene trend analysis also highlighted significant expression differences in *CTSB*, *LMNA*, and *SLC7A5* in immune-metabolic pathways, suggesting that these genes might contribute to GDM by modulating immune responses and metabolic processes.

CTSB shows a positive MR association with GDM risk, with no significant differential expression observed in human placentas, but increased expression in GDM mouse placentas. These discrepancies may be attributed to species-specific, tissue-specific, or context-specific regulation of *CTSB* expression. For instance, species differences between humans and mice could lead to divergent regulatory mechanisms. Additionally, tissue-specific regulation might affect *CTSB* expression differently in human placental tissues compared to the mouse placenta. Another potential factor contributing to these differences is the severity of hyperglycemia, which is generally more pronounced in animal models of GDM compared to human cases.

In summary, this study combined single-cell sequencing, Mendelian randomization, and immune cell infiltration analyses to elucidate the complex interactions among immune cells, key genes, and signaling pathways in GDM. Our findings provide a deeper understanding of the pathophysiology of GDM and offer potential molecular targets and therapeutic strategies for early diagnosis and personalized treatment. However, several limitations should be considered. First, because we used European ancestry-based GWAS data, the generalizability of our results may be limited by population structure. Second, although we identified several potential key genes with causal associations to GDM, functional validation of these genes remains necessary through experimental studies. Additionally, we relied on whole-blood cis-eQTLs from the eQTLGen consortium, which may not capture all relevant placental regulatory effects. This tissue mismatch may affect causal interpretation, and future research using placental-specific eQTLs or direct placental transcriptomic data would be important to address this limitation. Future research can further validate the relationship between these genes and GDM in large, multi-ethnic cohorts and explore their clinical applications.

By integrating single-cell transcriptomics, Mendelian randomization, and analyses of the immune microenvironment, this study delineates the cellular and molecular landscape of gestational diabetes mellitus. We identified *CTSB*, *LMNA*, and *SLC7A5* as robust candidate genes with causal and transcriptomic evidence and linked them to immune-metabolic pathways and altered immune cell infiltration. These findings advance understanding of GDM pathophysiology and provide a foundation for the development of biomarkers, targeted therapies, and precision interventions in pregnancy care.

## Data Availability

The raw data supporting the conclusions of this article will be made available by the authors, without undue reservation.
