# Supplementary figures and images for: Integrative multi-omics analysis reveals cellular and molecular insights into gestational diabetes mellitus
Source: Front Mol Biosci. 2026 Feb 18;13:1706588. doi: 10.3389/fmolb.2026.1706588 (PMC12957148; doi:10.3389/fmolb.2026.1706588)

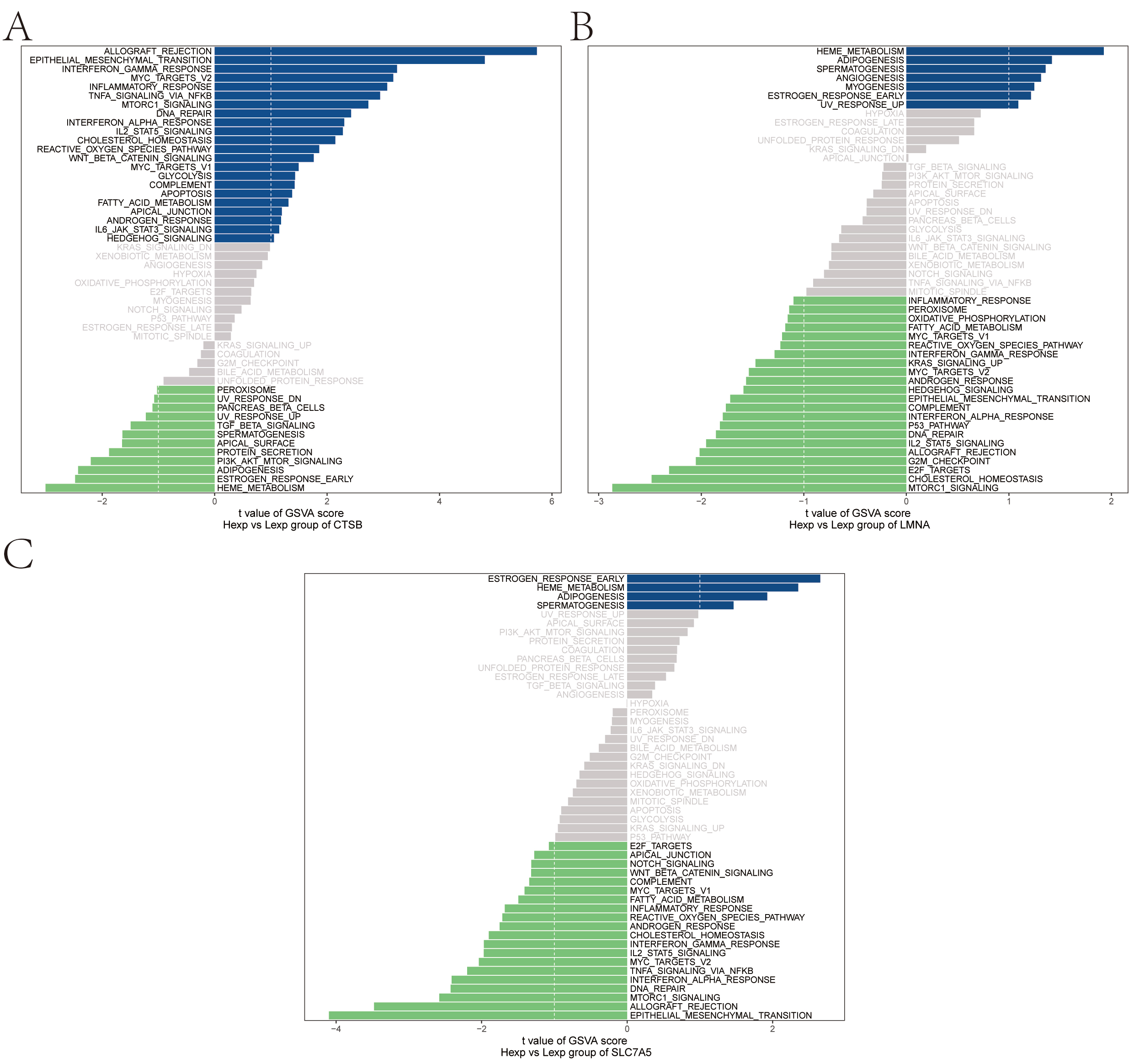

Supplement: Supplementary file 1 [file Image3.jpeg]

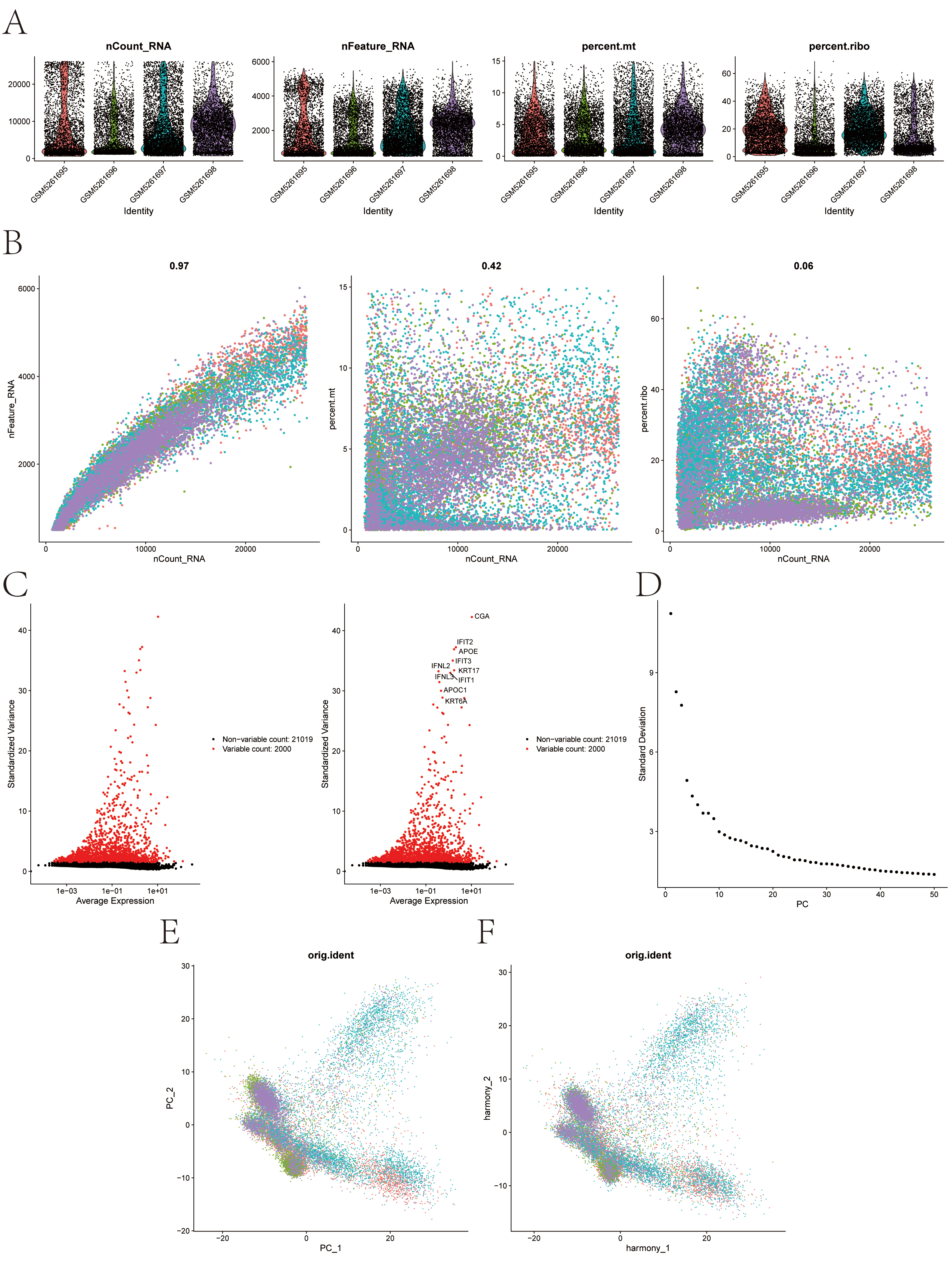

Supplement: Supplementary file 3 [file Image1.jpeg]

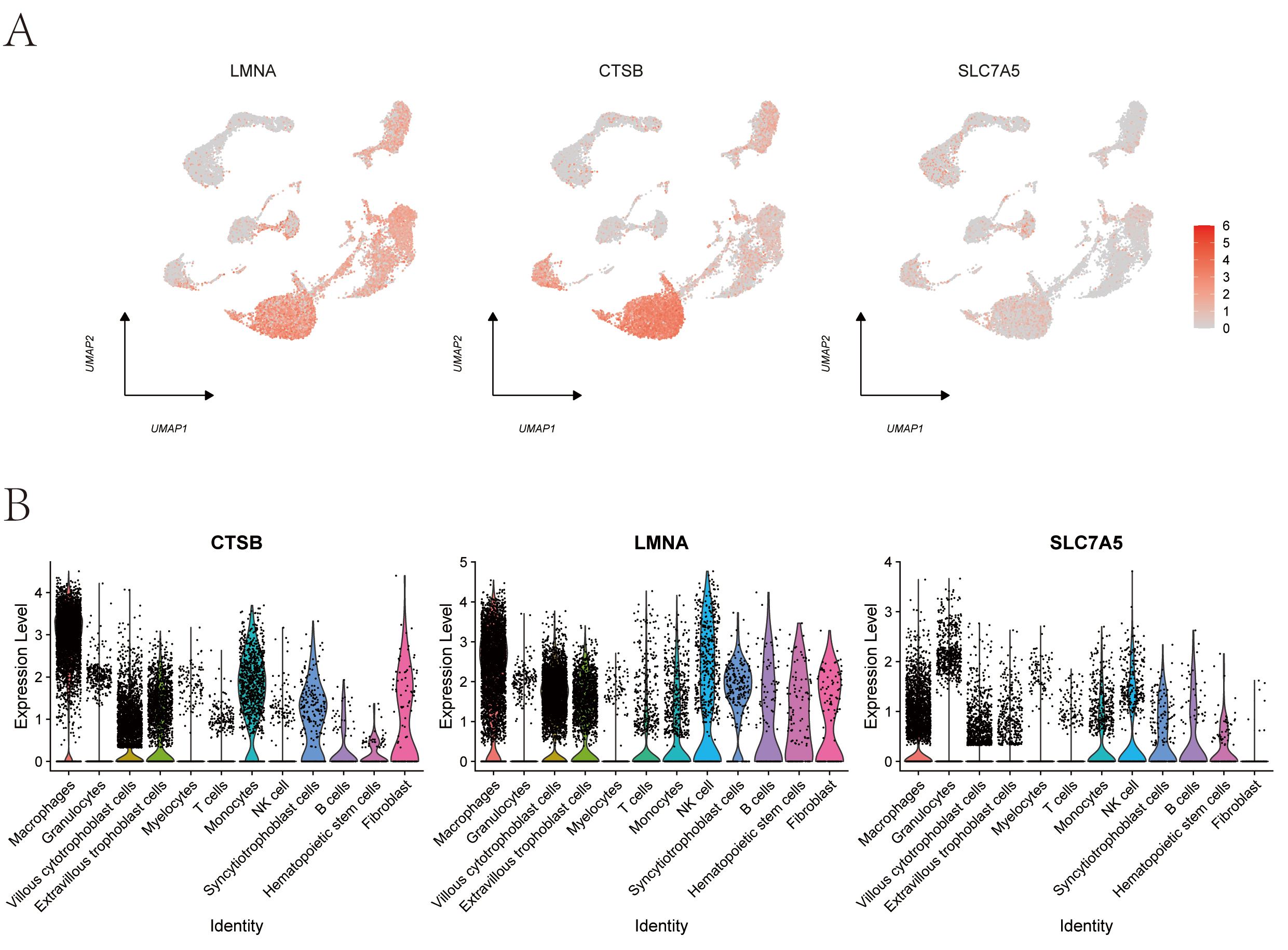

Supplement: Supplementary file 4 [file Image4.jpeg]

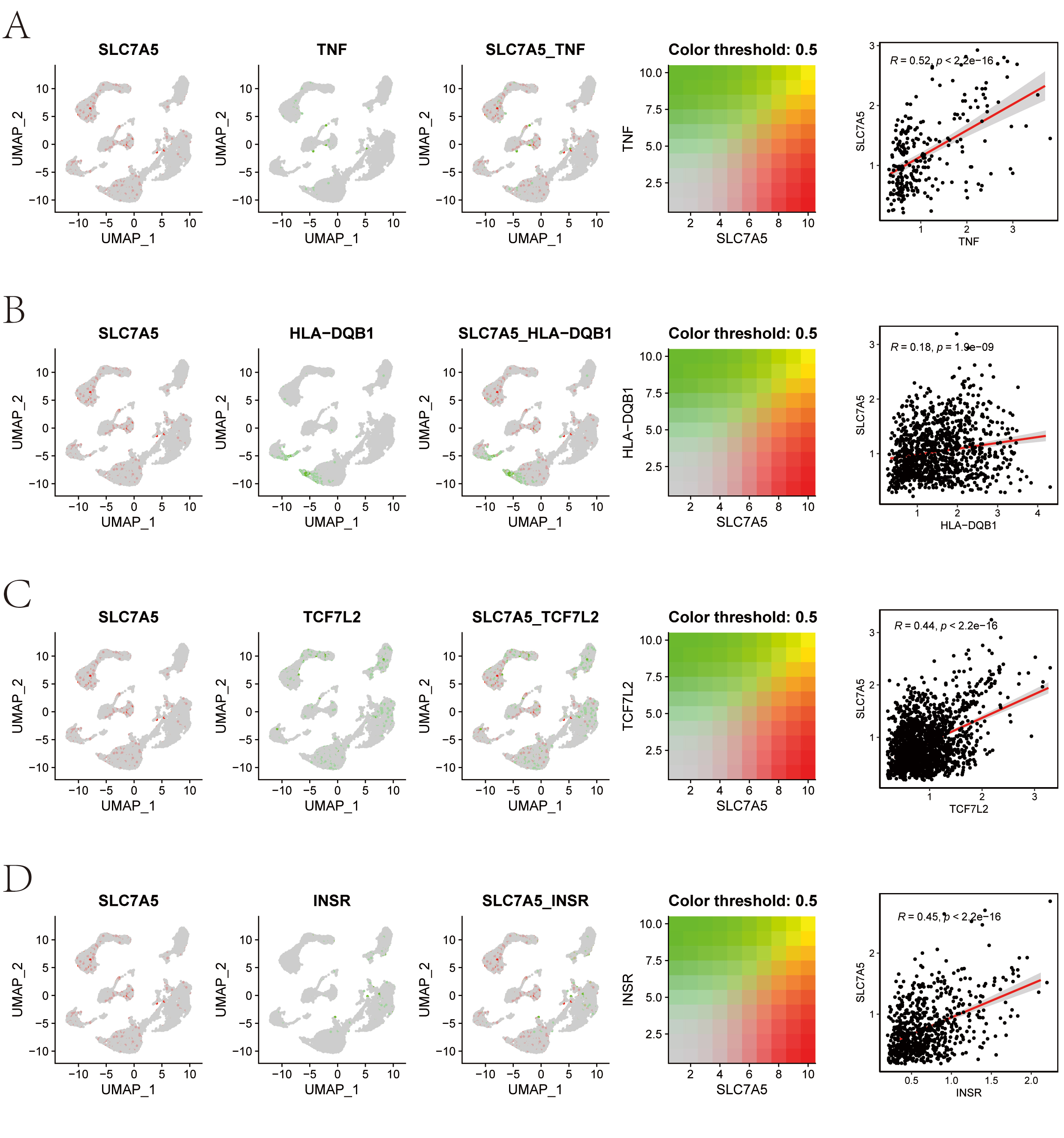

Supplement: Supplementary file 5 [file Image7.jpeg]

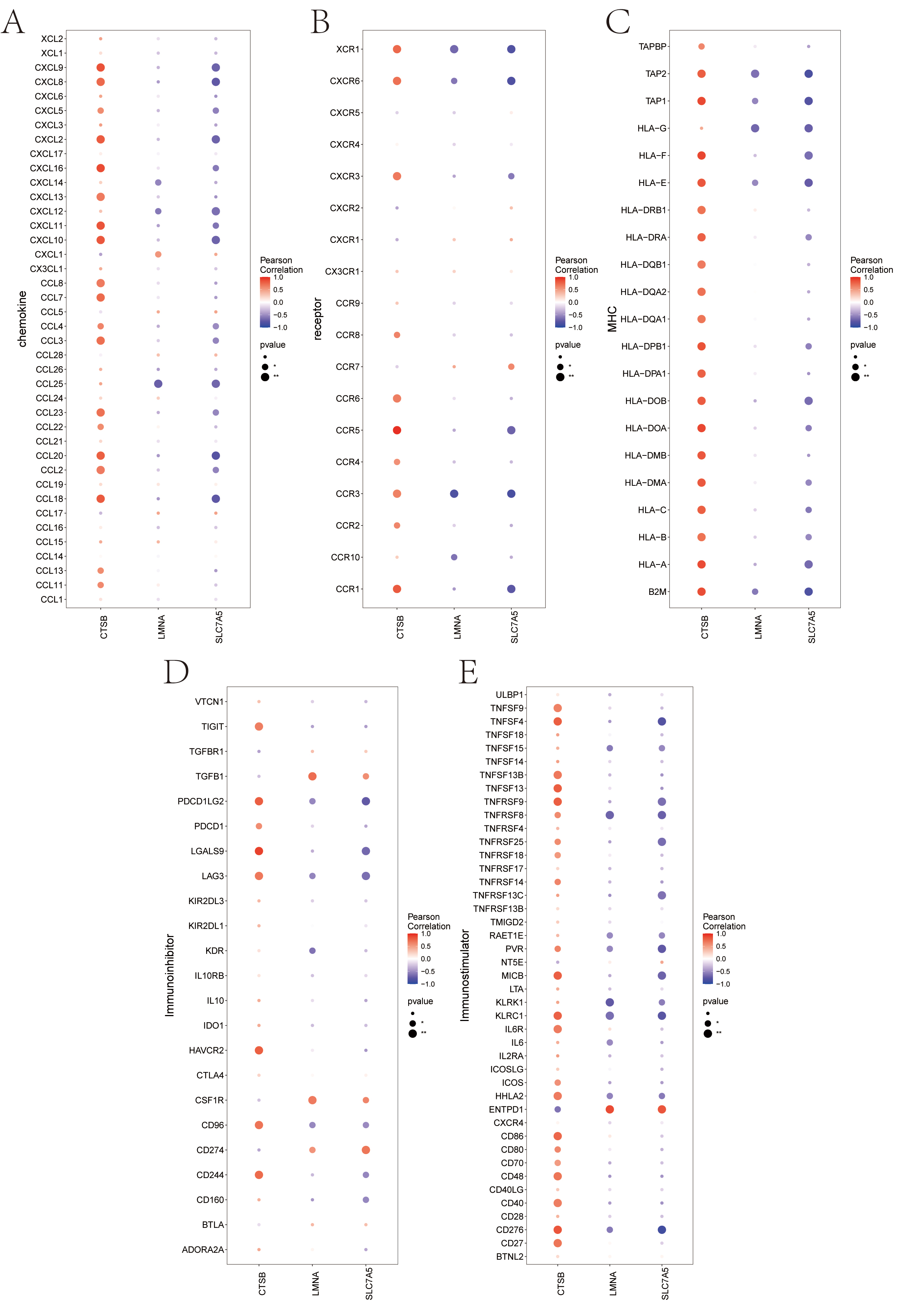

Supplement: Supplementary file 6 [file Image2.jpeg]

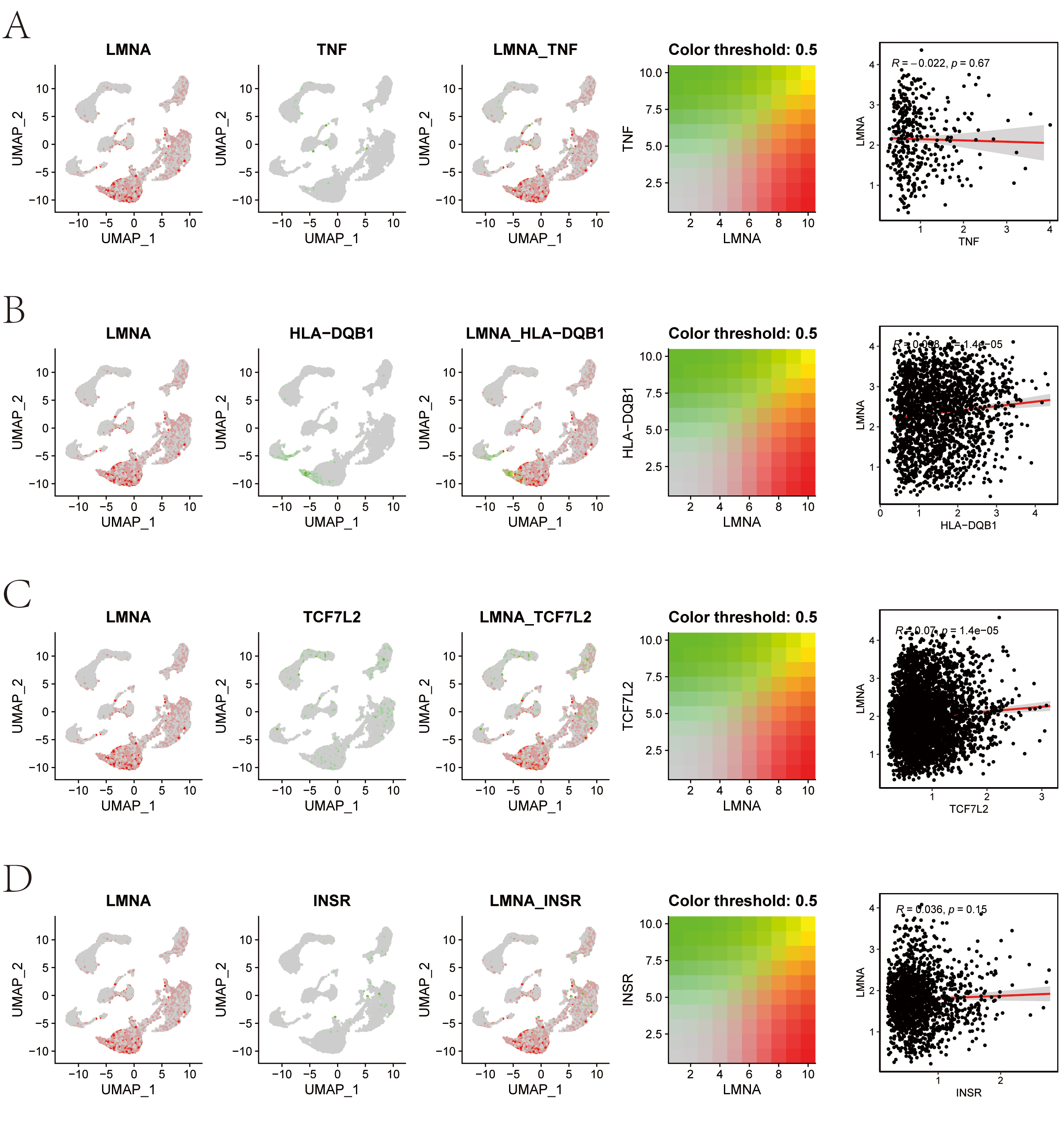

Supplement: Supplementary file 7 [file Image5.jpeg]

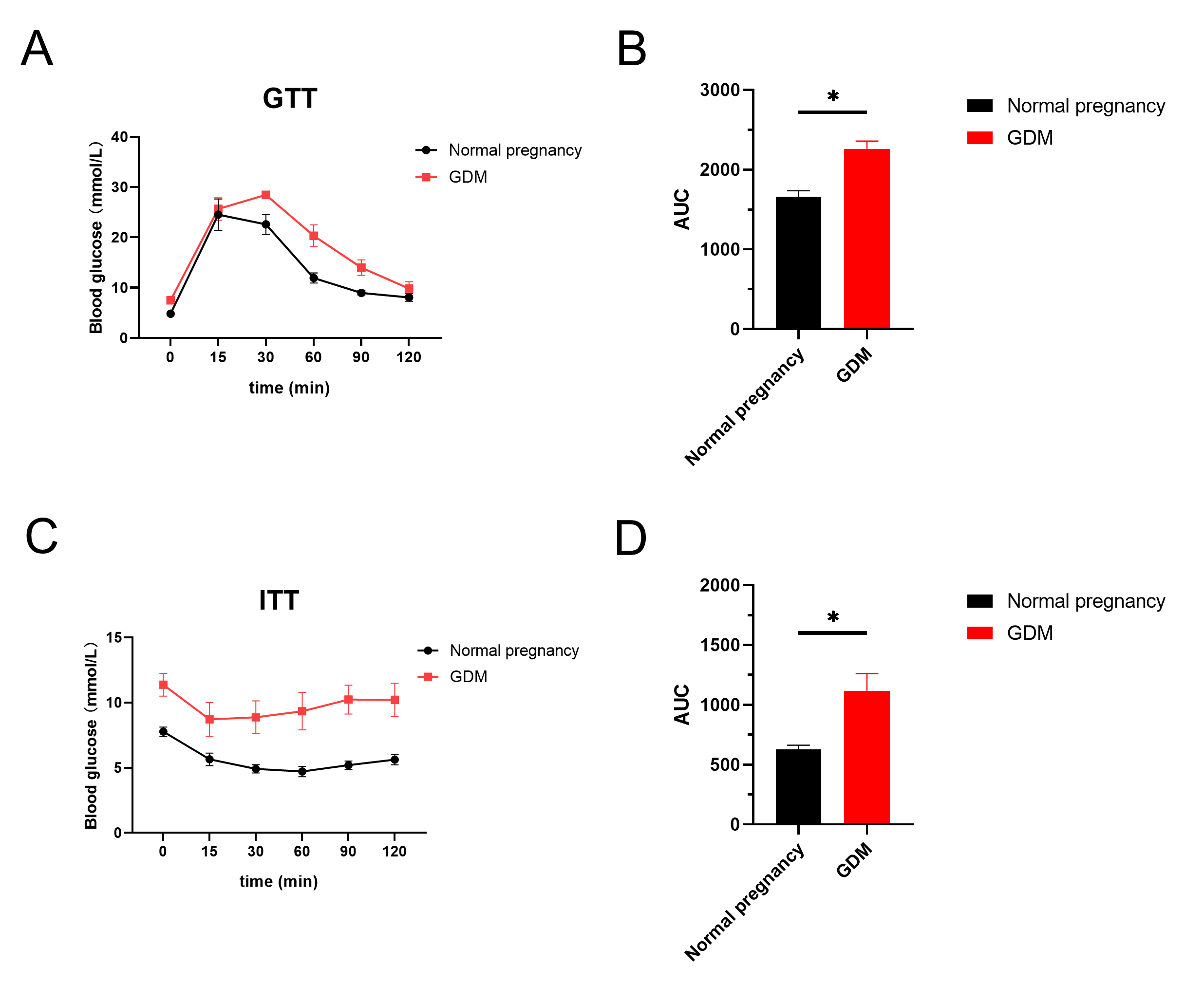

Supplement: Supplementary file 9 [file Image8.jpeg]

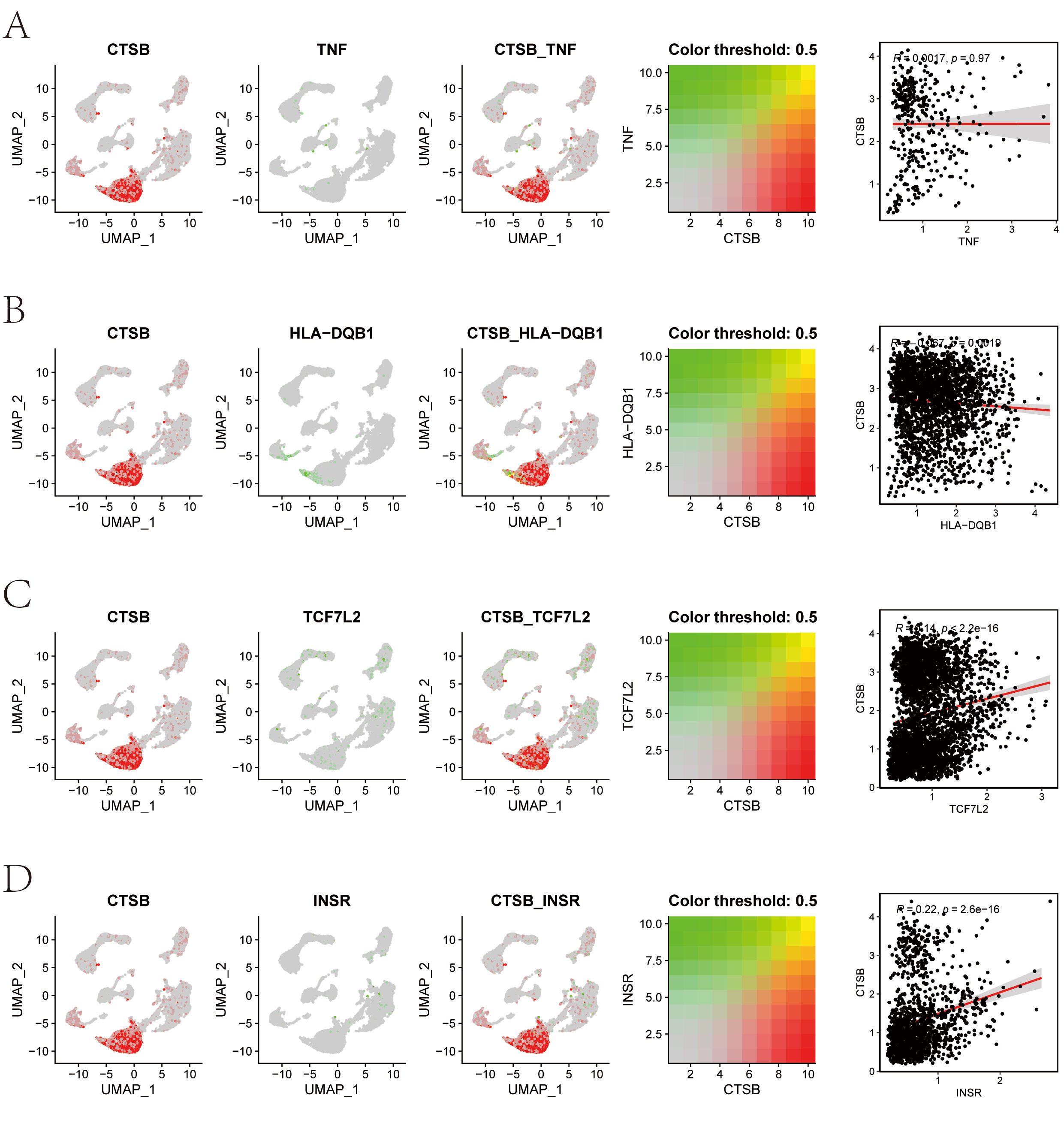

Supplement: Supplementary file 10 [file Image6.jpeg]
