# Supplementary material for: Integrative multi-omics analysis reveals cellular and molecular insights into gestational diabetes mellitus
Source: Front Mol Biosci. 2026 Feb 18;13:1706588. doi: 10.3389/fmolb.2026.1706588 (PMC12957148; doi:10.3389/fmolb.2026.1706588)
Supplement: Supplementary file 2 [file Table1.docx]

|  | Table S1. PCR primer sequences. |  |
| --- | --- | --- |
| **Gene** | **Forward (5***′* **-3***′* **)** | **Reverse (5***′* **-3***′* **)** |
| H-SLC7A5 | GGCATCGGCTTCACCATCATCC | GGCATCGGCTTCACCATCATCC |
| H-LMNA | GTACGGCTCTCATCAACTCCACTG | TCCTCATCCTCGTCGTCCTCAAC |
| H-CTSB | CTGCTGGCTGTAATGGTGGCTATC | GGGAGGGATGGAGTACGGTCTG |
| H-β-Actin | CATGTACGT TGCTATCCAGGC | CTCCTTAATGTCACGCACGAT |
| M-Slcya5 | GTGTGCGGCGTCTTCTCCATC | ACCTCCAGCATGTAGGCGTAGTC |
| M-Lmma | TCCCACCGAAGTTCACCCTA | TGGAGTTGATGAGAGCGGTG |
| M-Ctsb | GGCAGGCTGGACGCAACTTC | CTATGTCCTCACCGAACGCAACC |
| M-β-Actin | CAGCCTTCCTTCTTGGGTATG | GGCATAGAGGTCTTTACGGATG |
| H: human; M: mouse. | | |
