# Supplementary material for: Integrative multi-omics analysis reveals cellular and molecular insights into gestational diabetes mellitus
Source: Front Mol Biosci. 2026 Feb 18;13:1706588. doi: 10.3389/fmolb.2026.1706588 (PMC12957148; doi:10.3389/fmolb.2026.1706588)
Supplement: Supplementary file 8 [file Table2.docx]

| Table S2. Basic characteristics of the enrolled pregnant women | | | |
| --- | --- | --- | --- |
| **Sample Information** | **Control** | **GDM** | ***P*** |
| Age (yr.) | 34.13±4.42 | 37.52±5.42 | 0.216 |
| Pregnancy History (no.) |  |  |  |
| Primipara | 6 | 4 | 0.608 |
| Multipara | 2 | 4 |  |
| Pre-pregnancy BMI (kg/㎡) | 20.31±2.27 | 21.92±2.57 | 0.268 |
| Pre-delivery BMI (kg/㎡) | 25.68±3.19 | 26.59±2.28 | 0.718 |
| OGTT-0h (mmo1/L) | 4.21±0.33 | 4.32±0.29 | 0.609 |
| OGTT-1h (mmo1/L) | 7.60±1.64 | 9.83±0.68 | 0.004* |
| 0GTT-2h (mmo1/L) | 5.85±1.19 | 8.83±0.69 | ＜0.001* |
| Gestational Week (wk.) | 38.68±0.74 | 38.59±0.85 | 0.974 |
| wk.:week; yr.:year; no.:number; BMI: body mass index; OGTT: oral glucose tolerance test. *: P＜0.05. | | | |
